# Missing the vulnerable—Inequalities in social protection in 13 sub-Saharan African countries: Analysis of population-based surveys

**DOI:** 10.1371/journal.pgph.0002973

**Published:** 2024-07-02

**Authors:** David Chipanta, Silas Amo-Agyei, Lucas Hertzog, Ahmad Reza Hosseinpoor, Michael Smith, Caitlin Mahoney, Juan Gonzalo Jaramillo Meija, Olivia Keiser, Janne Estill

**Affiliations:** 1 United Nations Joint Programme on HIV/AIDS (UNAIDS), Windhoek, Namibia; 2 The University of Manchester, Manchester, United Kingdom; 3 United Nations High Commissioner for Refugees, Geneva, Switzerland; 4 Faculty of Health Sciences, WHO Collaborating Centre for Climate Change and Health Impact Assessment, School of Population Health, Curtin University, Perth, Australia; 5 Department of Data and Analytics, World Health Organization, Geneva, Switzerland; 6 Nutrition Division, World Food Programme, Rome, Italy; 7 Social Protection Unit, World Food Programme, Rome, Italy; 8 Institute of Global Health, University of Geneva, Geneva, Switzerland; McGill University, CANADA

## Abstract

We assessed socioeconomic inequalities in social protection coverage among the public, men and women living with the human immunodeficiency virus (MLHIV, WLHIV), and adolescent girls and young women (AGYW). We used population-based data from Cameroon, Côte d’Ivoire, Ethiopia, Eswatini, Kenya, Lesotho, Malawi, Namibia, Rwanda, Tanzania, Uganda, Zambia, and Zimbabwe. We constructed concentration curves (CC) and computed concentration indices (CIX) for each country and population group. A CC represents the cumulative percentage of social protection coverage plotted on the y-axis against the cumulative proportion of the population—ranked by socioeconomic status from the poorest to the richest—on the x-axis. The CIX quantifies the concentration of social protection coverage among the poor or the rich. The sample size ranged from 10,197 in Eswatini to 29,577 in Tanzania. Social protection coverage among the public varied from 5.2% (95% Confidence Interval 4.5%–6.0%) in Ethiopia to 39.9% (37.0%–42.8%) in Eswatini. It ranged from 6.9% (5.7%–8.4%) MLHIV in Zambia to 45.0% (41.2–49.0) among WLHIV in Namibia. Among AGYW, it varied from 4.4% (3.6–5.3) in Ethiopia to 44.6% (40.8–48.5) in Eswatini. Socioeconomic inequalities in social protection coverage favored the poor in 11/13 countries surveyed. It favored the rich in Cameroon and was undefined in Côte d’Ivoire. The CIX in these 11 countries ranged from −0.080 (p = 0.002) among the public in Malawi to −0.372 (p< 0.001) among WLHIV in Zimbabwe. In 8 of these 11 countries, ≥15% of people from the poorest households reported receiving social protection. Only in countries with higher levels of social protection coverage did most people from the poorest households achieve high coverage. Social protection coverage was low and favored the poor. Pro-poor social protection is insufficient to reach the poor. Research is required to reach the poorest households with social protection in Africa.

## 1 Introduction

Inequality in access to social services is an urgent global concern, with the alarming gap between extreme wealth and poverty reaching unprecedented levels and thrusting billions of people into hardship, including hunger [1,2]. Inequality is multifaceted, spanning race, ethnicity, income, wealth, and gender. Gender inequalities are deeply entrenched and intersect with other forms of inequality [3,4]. Generally, equitable communities enjoy robust social cohesion, low crime rates, high levels of trust, life satisfaction, durable peace, political stability, and economic growth, in contrast to their inequitable counterparts [3,5─^7^]. Conversely, high inequalities can undermine a nation’s capacity to prevent, respond, and adapt to emergencies, including infectious diseases [3,7]. Therefore, addressing inequalities is an imperative objective of the Sustainable Development Goals (SDGs). SDG 10–Reducing Inequalities—committed member states to reducing inequalities by promoting the inclusion of all population groups in socioeconomic and political spheres by 2030 [8]. Despite the increasing global focus on inequalities, current trajectories show that the world is unlikely to meet even 10% of the full targets under SDG 10 by 2030 [9]. People residing in the Global South, particularly in sub-Saharan Africa, bear the impact of the failure to achieve SDG10 [9].

Sub-Saharan Africa’s disproportionate burden from failing to meet SDG 10 stems largely from the existing social and economic vulnerabilities in the region. The region houses some of the world’s poorest nations, where inequalities are not merely prevalent but are escalating due to insufficient social protection systems and economic instability. This vulnerability is compounded by high rates of infectious diseases, political instability, and low level of development, which hinder effective responses to socioeconomic challenges. Without significant progress on reducing inequalities, sub-Saharan Africa risks entrenching a cycle of poverty and exclusion that undermines long-term development and social stability, thereby magnifying the adverse impacts of global inequality.

The focus of this study on PLHIV and AGYW is warranted given the distinct vulnerabilities these groups face in sub-Saharan Africa. PLHIV often experience heightened socioeconomic disparities due to stigma, reduced work capacity, and increased medical expenses, which can limit their access to social protection programs. Similarly, AGYW are disproportionately affected by HIV infection, early pregnancy, gender-based violence, and lower educational attainment, which exacerbate their economic and social vulnerabilities. Understanding the effectiveness of social protection for these specific groups is crucial for devising targeted interventions that address their unique barriers to accessing social services.

Social protection programs can accelerate progress toward achieving SDG 10. Social protection is defined as policies and programs that help individuals and societies manage risk and uncertainty, protect them from poverty and inequality, and allowing them to access economic opportunity [10]. Social protection reduces poverty, inequality, and the prevalence of ill health; fostering gender equality; and stimulating inclusive economic growth [11─^14^]. Social protection programs that cater to the poorest populations can alleviate inequality [15]. Such programs are pro-poor. They prioritize the most impoverished and vulnerable people, including children, women, persons with disabilities, and the elderly [11,16–18]. However, research investigating socioeconomic inequalities in receiving social protection in sub-Saharan Africa is limited. We assessed socioeconomic inequalities in receiving social protection among the general population, people (women and men) living with HIV (PLHIV), and adolescent girls and young women (AGYW). We used population-based impact assessment survey data from 13 sub-Saharan African countries. Our hypothesis was that social protection was pro-poor, focused on people from poor households who are considered most vulnerable and deserving of access to it.

## 2 Materials and methods

We analyzed SDG Indicator 1.3.1, defined as the proportion of the population receiving at least one social protection benefit from any source, as the main outcome indicator. UNAIDS earmarked this indicator as a target to measure the coverage of social protection for people living with, at risk of, or affected by HIV [19]. The target aims to ensure that by 2025, 45% of people living with, at risk of or affected by HIV have access to social protection benefits [19]. We examined inequalities in receiving social protection within the preceding 12 months of the survey interview by the general population, men and women living with HIV (MLHIV and WLHIV), and AGYW. We also assessed whether social protection in the participating countries reached the poorest households measured based on living standards including household assets.

We analyzed the population HIV impact assessment (PHIA) survey data for countries with data on social protection receipt by the general population, MLHIV and WLHIV, and AGYW from 13 countries. These countries surveyed between 2015 and 2019 were Cameroon, Côte d’Ivoire, Ethiopia, Eswatini, Kenya, Lesotho, Malawi, Namibia, Rwanda, Tanzania, Uganda, Zambia, and Zimbabwe (see specific survey year per country in the S1 Table. The PHIA surveys collected a range of health and socio-demographic data to evaluate the impact of HIV programs in the countries supported by the United States President’s Emergency Plan for AIDS Relief. We used the Household, Adult Interview, and Adult HIV Biomarker datasets. In participating households, a household questionnaire was administered to the household head, who indicated all individuals living in the household and provided information on the household, such as assets, living standards, and access to social protection benefits. Individual questionnaires were then administered to eligible and consenting adults aged 15 or older in the household. The Adult HIV Biomarker data set contained the HIV test results of all adults and adolescents aged 15 or older who completed an individual interview and consented or agreed to provide blood samples for HIV testing. The interviews assessed wealth, education level, and other socio-demographic characteristics at the individual and household levels. They also included questions about external economic support. In addition, the questions identified AGYWs aged 15–24 years. We obtained the PHIA data sets from the PHIA Project website at https://phia-data.icap.columbia.edu/. We also conducted a review of the literature on social protection in the studied countries to support the writing of the paper including the Discussion section.

### 2.1 Variables and outcome descriptions

Our primary outcome was social protection, defined as a binary variable with value 1 if the respondent lives in a household receiving any form of external economic support within the previous 12 months of the survey. Social protection was derived from the PHIA household survey question “Has your household received any of the following forms of economic support in the last 12 months: assistance for school fees, material support for education, food assistance, support for income generation, social pensions, and cash transfers, including pensions, disability, and child grants?

We classified a respondent as HIV-positive if the respondent self-reported an HIV-positive test result, and their screening and confirmatory HIV biomarker test were also positive. Respondents with a positive screening result but negative confirmatory result were classified as indeterminate and excluded from the analysis. Respondents self-reporting HIV-negative and/ or with a negative screening result but a positive confirmatory test result were classified as HIV-positive and included in the analysis. AGYW were defined as females aged 15–24 years, men as males 15 years and older and women as females 15 years and older. Other explanatory variables used in the analysis included HIV prevalence, age, sex, marital status, household size, residence location (rural versus urban), employment status, education, wealth quintiles, and region of residence. Wealth quintiles ranged from Quintile one (Q1), that is, the bottom 20% of households to Q5, the wealthiest 20% of households. The variables are defined in S2 Table.

The study sample included women and men aged 15–59 years who were interviewed. Any individual with a missing information on the sex variable was excluded from the analysis. Household wealth was evaluated via a composite measure reflecting living standards, based on asset ownership, which included items such as television sets, refrigerators, water access, and roofing. Region reflected the subregion of a country and was included in the analyses to account for subregional variation in access to social protection.

### 2.2 Analysis

#### 2.2.1 Measuring the level of social protection coverage

We used the *surveymeans* procedure to determine the weighted proportion of persons who reported receiving any social protection benefit for each country and population group – the overall population in each country aged 15 to 59, MLHIV, WLHIV and AGYW. Survey weights accounting for nonresponse using Chi-squared automatic interaction detector analysis, noncoverage, and the probability of selection were applied. We used individual interview weights in the analysis of the data. Variances and 95% confidence intervals (CIs) were estimated using the corresponding jackknife replicate weights [20].

#### 2.2.2 Measuring inequality in social protection coverage

We used two methodologies to examine income-related inequality in receiving social protection. First, we constructed concentration curves for receiving social protection for each subpopulation within each country. A concentration curve represents the cumulative percentage of a variable of interest—in this study, social protection —plotted on the y-axis against the cumulative proportion of the population—ranked by socioeconomic status from the poorest to the richest—on the x-axis [21]. The concentration curve coincides with the 45° line, known as the line of equality, when every individual receives the same value of the variable of interest. A concentration curve lying above (below) the line of equality signifies that the variable of interest is concentrated among the poor (rich). The degree of pro-poor (pro-rich) inequality increases as the curve diverges further above (below) the line of equality. In this study, we defined pro-poor social protection by the concentration curve of receiving social protection above the line of equality.

In the second approach, we computed the Concentration Index (CIX). The CIX encapsulates the information conveyed by the concentration curve, quantifying the socioeconomic inequalities associated with the variable of interest—social protection. The CIX is twice the area between the concentration curve and the line of equality, equating to zero in the absence of economic-related inequality [21]. A negative (positive) CIX value signifies that the curve lies above (below) the line of equality, indicating a disproportionate concentration of the variable of interest among the poor (rich). A zero CIX value can also occur if the curve intersects the line of equality and the areas above and below the equality line offset each other. In standard practice, CIX is interpreted in conjunction with the concentration curve. We conducted all analyses using Stata version 18.

#### 2.2.3 Ethics statement

PHIA survey administration follows international scientific research standards in human subjects, including protecting respondents’ privacy and confidentiality of information. Each country’s PHIA survey report provides details of the survey design, sampling procedure, protection of the privacy and confidentiality of information, and obtaining informed consent (S1 Table).

Ethics and regulatory bodies, including ministries of health and institutional review boards, approved the PHIA survey protocols, consent forms, questionnaires, and other survey documents in each country. The institutional review boards of Columbia University Medical Center, Westat, and the Centers for Disease Control also reviewed and approved the survey documents.

This study did not require ethical clearance because the data were de-identified. It can be accessed by registering at the PHIA Project website at PHIA Data Manager (columbia.edu)

## 3. Results

The sample size ranged from 10,197 in Eswatini to 29,577 in Tanzania, with median ages ranging between 27 years (interquartile range, IQR, 20–37 in Uganda) to 32 years (IQR 25–41 in. Kenya) (Table 1). HIV prevalence was lowest in Côte d’Ivoire (2.7%, 95% CI (2.4%–3.1%)) and highest in Eswatini (27.9%, 26.5%–29.3%). Women comprised 60% or more of people living with HIV in the surveyed countries (S3 Table).

**Table 1:** Survey weighted sample descriptive statistics by country (PHIA 2015-2019). The results are reported as (percentages with, sample size, 95% confidence intervals and absolute numbers unless otherwise indicated).

	Cameroon (N = 26039)	Côte d’Ivoire (N = 18339)	Eswatini (N = 10197)	Ethiopia (N = 18466)	Kenya (N = 23536)	Lesotho (N = 12842)	Malawi (N = 19092)
HIV prevalence	3.6 (3.3 – 4.0) 924	2.7 (2.4 – 3.1) 417	27.9 (26.5 – 29.3) 2776	3.0 (2.6 – 3.4) 588	5.8 (5.4 – 6.3) 1387	25.6 (24.7 – 26.5) 3192	10.5 (9.9 – 11.2) 2155
Sex							
Male	49.1 (49.0–49.2) 11827	51.3 (51.2–51.4) 9145	45.5 (45.5–45.6) 4377	50.0 (49.9–50.1)7735	47.5 (47.1–47.9) 9468	50.1 (50.0–50.1) 5339	48.5 (48.5–48.5) 8002
Female	50.9 (50.8–51.0) 14212	48.7 (48.6–48.8) 9194	54.5 (54.4–54.5) 5820	50.0 (49.9–50.1) 11731	52.5 (52.1–52.9) 14068	49.9 (49.9–50.0) 7503	51.5 (51.5–51.5) 11090
Age [Years] (IQR)	29 (21–39)	29 (22–39)	28 (21–38)	28 (21–38)	32 (25–41)	30 (22–40)	28 (20–38)
15-24	36.6 (36.6–36.7) 9333	34.3 (34.2–34.4) 6199	37.2 (37.2–37.3) 3785	35.7 (35.6–35.8) 7882	21.9 (21.4–22.5) 4513	34.1 (34.0–34.2) 4403	39.8 (39.8–39.9) 7166
25-34	28.1 (28.0–28.2) 7474	31.2 (31.2–31.3) 5387	29.0 (29.0–29.1) 2843	31.3 (31.2–31.3) 5982	35.0 (34.7–35.2) 7857	29. 9 (29.8–29.9) 3640	27.6 (27.6–27.6) 5489
35-44	19.4 (19.4–19.5) 4928	19.2 (19.2–19.3) 3818	18.7 (18.6–18.7) 1820	19.4 (19.3–19.4) 3288	23.8 (23.5–24.0) 5770	19.0 (18.9–19.0) 2387	18.2 (18.2–18.2) 3690
45-54	11.8 (11.7–11.8) 3084	11.6 (11.6–11.6) 2221	11.3 (11.3–11.3) 1256	10.4 (10.3–10.4) 1730	14.5 (14.4–14.7) 3911	12.1 (12.0–12.1) 1593	10.8 (10.8–10.8) 2050
55+	4.0 (4.0–4.1) 1220	3.6 (3.6–3.6) 714	3.8 (3.8–3.9) 493	3.3 (3.3–3.4) 584	4.8 (4.8–4.9) 1485	5.0 (4.9–5.0) 819	3.6 (3.6–3.6) 697
Residence type							
Rural	47.4 (43.4–51.4)14731	37.4 (33.8–41.2) 8762	72.0 (69.7–74.2) 7835		60.5 (58.5–62.5) 14548	58.8 (56.4–61.2) 7774	79.9 (77.0–82.5) 11827
Urban	52.6 (48.6–56.6) 11308	62.6 (58.8–66.2) 9577	28.0 (25.8–30.3) 2362	100.0(89.9 -100.1)18466	39.5 (37.5–41.5) 8988	41.2 (38.8–43.6) 5068	20.1 (17.5–23.0) 7265
Marital status							
Single	17.1 (16.4–17.7) 4402	8.8 (8.0–9.6) 1482	12.2 (11.5–13.0) 1290	14.8 (14.1–15.5) 3124	14.7 (14.0–15.3) 3670	17.0 (16.3–17.7) 2371	12.0 (11.3–12.7) 2441
Married	82.9 (82.3–83.6) 21637	91.2 (90.4–92.0) 16857	87.8 (87.0–88.5) 8907	85.2 (84.5–85.9) 16342	85.3 (84.7–86.0) 19866	83.0 (82.3–83.7) 10471	88.0 (87.3–88.7) 16651
Household size (n)							
1 to 3	23.2 (21.9–24.6) 5823	25.7 (24.2–27.2) 4752	31.2 (29.2–33.3) 3146	39.5 (38.0–41.1) 8075	32.0 (30.5–33.5) 6774	40.5 (39.1–42.0) 5259	25.2 (23.9–26.5) 5075
4 to 6	35.8 (34.4–37.3) 9022	34.8 (32.6–37.0) 6308	37.4 (35.7–39.1) 3795	45.0 (43.7–46.3) 8361	44.8 (43.5–46.0) 10649	43.3 (41.8–44.8) 5543	50.8 (49.3–52.2) 9597
>7	40.9 (38.9–43.1) 11194	39.6 (37.0–42.2) 7279	31.4 (29.2–33.7) 3256	15.5 (14.0–17.0) 3030	23.2 (22.0–24.6) 6113	16.1 (14.8–17.6) 2040	24.0 (22.6–25.5) 4420
Employment status							
Not employed	45.1 (43.8–46.5) 12218	52.7 (51.1–54.3) 9962	56.7 (55.2–58.2) 5975	52.2 (50.8–53.7) 10996	42.4 (41.1–43.8) 11382	60.9 (59.6–62.1) 8156	70.4 (69.3–71.5) 13156
Employed	54.9 (53.5–56.2) 13821	47.3 (45.7–48.9) 8377	43.3 (41.8–44.8) 4222	47.8 (46.3–49.2) 8470	57.6 (56.2–58.9) 12154	39.1 (37.9–40.4) 4686	29.6 (28.5–30.7) 5936
Education level							
Not educated	13.0 (11.9–14.3) 4695	40.8 (38.5–43.1) 7984	3.5 (3.0–4.0) 384	11.3 (10.3–12.4) 2318	7.5 (6.7–8.3) 2650	5.0 (4.5–5.5) 620	8.6 (8.0–9.3) 1521
Primary	26.2 (24.9–27.5) 7358	25.6 (24.3–26.9) 4904	25.9 (24.7–27.3) 2795	35.1 (33.7–36.6) 6821	49.5 (48.2–50.8) 12002	39.8 (38.2–41.4) 5247	63.2 (61.7–64.7) 10894
Secondary	33.5 (32.5–34.6) 8146	26.9 (25.4–28.5) 4539	59.9 (58.4–61.3) 6032	29.1 (28.0–30.2) 5706	30.9 (29.7–32.1) 6341	44.6 (43.2–46) 5719	25.3 (23.9–26.7) 5689
Higher	27.3 (25.4–29.3) 5840	6.7 (5.5–8.2) 912	10.7 (9.4–12.2) 986	24.5 (22.7–26.3) 4621	12.1 (11.2–13.1) 2543	10.6 (9.6–11.7) 1256	2.9 (2.6–3.3) 988
Wealth quintiles:							
Q1: Poorest	19.9 (17.7–22.4) 7529	25.4 (22.0–29.1) 2867	20.2 (18.5–22.1) 2224	16.5 (14.4–18.9) 3379	19.9 (18.4–21.5) 6151	17.3 (15.5–19.3) 2485	15.2 (14.0–16.5) 2228
Q2	20.1 (17.8–22.7) 5776	19.1 (16.5–22.0) 3304	19.9 (18.2–21.7) 2139	17.6 (16.3–19.0) 3495	20.5 (19.3–21.8) 4937	18.9 (17.6–20.4) 2530	18.2 (16.9–19.5) 2725
Q3	21.3 (19.6–23.0)4864	18.4 (16.4–20.6) 4169	22.9 (20.8–25.1) 2374	19.8 (18.4–21.3) 3895	20.7 (19.5–21.8) 4867	20.1 (18.6–21.7) 2539	20.1 (18.9–21.4) 3040
Q4	18.9 (17.2–20.7) 4065	19.7 (17.8–21.7) 4508	18.0 (16.1–20.1) 1697	22.1 (20.2–24.0) 4212	20.2 (18.6–21.9) 4441	21.0 (19.6–22.5) 2592	22.1 (20.7–23.7) 3902
Q5: Wealthiest	19.8 (17.2–22.6) 3805	17.3 (14.5–20.6) 3491	19.0 (16.2–22.2) 1763	24.1 (21.8–26.5) 4485	18.8 (17.1–20.6) 3140	22.6 (20.7–24.8) 2696	24.4 (22.3–26.5) 7197

Q1 Quintiles 1: Poorest, Q5: Wealthiest. IQR Interquartile range ___ data set had no variable. Lesotho, we combined the population living in peri-urban areas with rural areas. Ethiopia data set only had an urban variable.

More than 60% of the respondents lived in rural areas in eight countries (i.e., Eswatini, Kenya, Lesotho, Malawi, Rwanda, Tanzania, Uganda, and Zimbabwe). In all countries surveyed, 80% or more of respondents were married or cohabiting, and 60% or more had at least four members. Of the respondents, 50%–70% were unemployed, except in Cameroon, Kenya, and Uganda, where less than 50% were unemployed. Up to 13% of the respondents had no formal education, except in Côte d’Ivoire, where 42% had no formal education. In general, the proportion of respondents from wealth quintile one (Q1), that is, the bottom 20% of households, was like those from Q2–Q5 (Table 1, Table 2).

**Table 2:** Survey weighted sample descriptive statistics by country (PHIA 2015-2019). The results are reported as (percentages with 95% confidence intervals and absolute numbers unless otherwise indicated).

	Namibia (N = 18009)	Rwanda (N = 29510)	Tanzania (N = 29577)	Uganda (N = 28212)	Zambia (N = 21138)	Zimbabwe (N = 21424)
HIV prevalence	12.5 (11.7 – 13.4) 2335	3.0 (2.6 – 3.3) 886	5.0 (4.7 – 5.4) 1707	6.3 (5.9 – 6.7) 1700	12.0 (11.3 – 12.6) 2447	14.1 (13.4 – 14.8) 3235
Sex						
Male	48.3 (48.2–48.3) 7967	48.1 (48.1–48.1) 13299	49.1 (49.1–49.1) 12867	47.4 (47.3–47.4) 12004	48.9 (48.9–49.0) 9104	47.7 (47.6–47.7) 8831
Female	51.7 (51.7–51.8) 10042	51.9 (51.9–51.9) 16211	50.9 (50.9–50.9) 16710	52. 6 (52.6–52.7) 16208	51.1 (51.0–51.1) 12034	52.3 (52.3–52.4) 12593
Age [Years] (IQR)	29 (21–40)	29 (21–39)	28 (20–39)	27 (20–37)	27 (20–38)	29 (21–38)
15-24	35.2 (35.1–35.2) 6081	35.7 (35.7–35.7) 11365	38.4 (38.4–38.5) 10704	43.4 (43.3–43.4) 11321	41.1 (41.0–41.2) 8043	37.8 (37.8–37.8) 7730
25-34	28.7 (28.6–28.7) 4871	28.9 (28.8–28.9) 8181	27.3 (27.3–27.4) 8127	26.8 (26.8–26.9) 7643	27.3 (27.2–27.3) 5736	28.4 (28.4–28.4) 5570
35-44	19.4 (19.3–19.4) 3657	19.5 (19.5–19.5) 5636	18.8 (18.8–18.8) 5875	16.5 (16.4–16.5) 4923	18.1 (18.1–18.2) 4146	19.6 (19.6–19.6) 4360
45-54	12.5 (12.5–12.6) 2450	11.1 (11.1–11.1) 3086	11.5 (11.5–11.5) 3671	10.1 (10.1–10.1) 3315	10.1 (10.1–10.2) 2418	10.1 (10.1–10.1) 2596
55+	4.3 (4.3–4.3) 950	4.8 (4.8–48) 1242	3.9 (3.9–4.0) 1200	3.3 (3.2–3.3) 1010	3.4 (3.3–3.4) 795	4.1 (4.1–4.2) 1168
Residence						
Rural	41.7 (39.4–44.0) 10146	79.3 (75.2–82.9) 21969	62.5 (58.7–66.2) 19601	71.3 (67.3–74.9) 20515	54.3 (50.8–57.6) 11911	64.0 (62.3–65.6) 14924
Urban	58.3 (56.0–60.6) 7863	20.7 (17.1–24.8) 7541	37.5 (33.8–41.3) 9976	28.7 (25.1–32.7) 7697	45.7 (42.4–49.2) 9227	36.0 (34.4–37.7) 6500
Marital status						
Single	18.7 (17.8–19.6) 3467	11.0 (10.6–11.5) 3250	13.4 (12.8–14.0) 4085	16.9 (16.3–17.5) 4866	11.4 (10.9–11.9) 2612	13.0 (12.4–13.6) 3111
Married	81.3 (80.4–82.2) 14542	89.0 (88.5–89.4) 26260	86.6 (86.0–87.2) 25492	83.1 (82.5–83.7) 23346	88.6 (88.1–89.1) 18526	87.0 (86.4–87.6) 18313
Household size (n)						
1 to 3	30.9 (29.5–32.4) 5294	15.2 (14.3–16.2) 4595	24.0 (22.7–25.4) 6749	19.5 (18.6–20.5) 5018	18.1 (17.1–19.2) 3836	27.8 (26.6–29.1) 6035
4 to 6	33.7 (32.1–35.4) 5783	44.3 (42.8–45.8) 12971	41.1 (39.8–42.4) 11799	35.7 (34.5–37.0) 9730	42.9 (41.8–44.1) 9176	49.4 (48.0–50.8) 10403
>7	35.4 (33.5–37.2) 6932	40.5 (38.6–42.4) 11944	34.9 (33.1–36.7) 11029	44.8 (43.2–46.3) 3464	38.9 (37.5–40.4) 8126	22.8 (21.4–24.2) 4986
Employment status						
Not employed	54 (52.8–55.3) 10329	59.5 (58.3–60.6) 17441	55.3 (54.2–56.4) 16829	47.0 (45.9–48.0) 13776	66.2 (65.0–67.4) 14295	59.8 (58.6–60.9) 13423
Employed	46.0 (44.7–47.2)7680	40.5 (39.4–41.7) 12069	44.7 (43.6–45.8) 12748	53.0 (52.0–54.1) 14436	33.8 (32.6–35.0) 6843	40.2 (39.1–41.4) 8001
Education level						
Not educated	6.7 (6.0–7.4) 1579	9.1 (8.6–9.6) 2502	12.9 (11.9–14.0) 4403	7.1 (6.6–7.6) 2486	4.9 (4.3–5.7) 1121	1.9 (1.7–2.1) 518
Primary	23.3 (22.2–24.5) 4994	61.6 (60.3–62.8) 17643	61.5 (60.4–62.6) 18231	55.7 (54.3–57.0) 16061	41.9 (40.2–43.6) 9164	25.1 (24.1–26.2) 6143
Secondary	58.0 (56.4–59.5) 9972	25.3 (24.2–26.3) 7921	20.3 (19.3–21.4) 5593	26.2 (25.3–27.2) 6819	44.8 (43.2–46.3) 9170	64.8 (63.6–66.0) 13314
Higher	12.0 (10.5–13.6) 1464	4.1 (3.6–4.7) 1444	5.3 (4.7–5.9) 1350	11.1 (10.2–12.0) 2846	8.4 (7.4–9.6) 1683	8.2 (7.2–9.3) 1449
Wealth quintiles						
Q1: Poorest	19.2 (17.8–20.7) 4610	18.7 (16.8–20.7) 5054	18.6 (16.4–21.1) 6155	20.5 (19.2–22.0) 7631	15.0 (13.6–16.5) 3357	19.2 (17.7–20.9) 4965
Q2	19.8 (18.0–21.8) 4122	19.0 (17.5–20.6) 5275	20.3 (18.7–21.9) 6154	19.6 (18.1–21.2) 5618	18.0 (16.6–19.5) 3931	19.6 (18.5–20.8) 4487
Q3	21.4 (19.4–23.5) 3827	19.9 (18.6–21.2) 5543	20.8 (19.3–22.4) 6583	19.7 (18.3–21.2) 5240	20.0 (18.3–21.8) 4274	19.2 (17.8–20.7) 4122
Q4	20.3 (18.2–22.6) 3077	20.8 (19.3–22.3) 5856	19.6 (17.8–21.6) 5510	19.8 (18.4–21.3) 4611	21.7 (19.6–24.0) 4526	19.7 (17.8–21.9) 3637
Q5: Wealthiest	19.3 (16.7–22.1) 2373	21.7 (19.3–24.4) 7782	20.7 (18.7–22.8) 5175	20.3 (18.1–22.7) 5112	25.2 (22.7–28.0) 5050	22.2 (20.1–24.4) 4213

Q1 Quintiles 1: Poorest, Q5: Wealthiest. IQR Interquartile range.

The proportion of the general population living in a household receiving any form of social protection varied from 5.2% (95% CI 4.5%–6.0%) in Ethiopia to 39.9% (37.0%–42.8%) in Eswatini. Among PLHIV households, the proportion receiving social protection varied from 6.9% (5.7%–8.4%) among MLHIV in Zambia to 45.0% (41.2–49.0) among WLHIV in Namibia. Among AGYW, the proportion varied from 4.4% (3.6–5.3) in Ethiopia to 44.6% (40.8–48.5) in Eswatini.

The proportion of the general population reporting receiving social protection from the poorest wealth quintile (Q1) ranged from 8.1% (6.4%–10.2%) in Cameroon to 56.2% (51.5%–60.7%) in Eswatini. Among the wealthiest quintiles (Q5), the proportion ranged from 3.6% (2.6%–5.0%) in Ethiopia to 19.7% (16.25–23.8%) in Namibia (Table 3, Table 4, Table 5). In general, 15% or less of the respondents from Q1 reported receiving social protection in eight countries (i.e., Cameroon, Côte d’Ivoire, Ethiopia, Kenya, Malawi, Tanzania, Uganda, and Zambia), with 10% or less in three countries (Cameroon, Côte d’Ivoire, and Ethiopia); 15%–20% in Rwanda, 30% in Zimbabwe, 40% in Lesotho, and more than 50% in Eswatini and Namibia.

**Table 3:** Survey weighted household social protection coverage for the general population, among people living with HIV (male and female), and adolescent girls and adolescent girls and young women by country (PHIA 2015-2019) (percent, 95% confidence interval).

	Cameroon				Côte d’Ivoire§			Eswatini			
	Gen pop	MLHIV	WLHIV	AGYW	Gen pop	WLHIV	AGYW	Gen pop	MLHIV	WLHIV	AGYW
Coverage	16.3 (14.7–18.0)	13.3 (8.9–19.5)‡	18.3 (14.9–22.4)	17.4 (15.4–19.7)	11.7 (10.3–13.2)	11.9 (7.9–17.4)‡	12.8 (10.8–15.1)	39.9 (37.0–42.8)	37.0 (32.6–41.5)	39.2 (35.6–43.0)	44.6 (40.8–48.5)
Residence Rural	15.0 (12.7–17.7)	---	21.3 (15.3–28.9)	15.3 (12.5–18.5)	11.7 (9.7–14.0)	---	9.8 (7.4- 12.9)	46.1 (43.1–49.2)	44.8 (39.7–50.1)	46.1 (41.9–50.4)	50.7 (46.6–54.7)
Urban	17.5 (15.4–19.7)	---	16.0 (12.1–20.9)	19.4 (16.4–22.9)	11.7 (9.9–13.7)	9.6 (5.6–16.0)‡	14.1 (11.5–17.1)	23.7 (17.5–31.3)	20.4 (14.6–27.8)	22.9 (17.4–29.5)	26.9 (18.3–37.8)
Employment status											
Not employed	13.2 (11.8–14.9)	---	11.6 (7.9–16.8)‡	16.2 (14.2–18.3)	12.2 (10.6–13.9)	14.6 (8.8–23.3)‡	13.3 (10.9–16.0)	43.9 (40.9–46.9)	43.0 (36.7–49.6)	42.2 (38.3–46.2)	45.9 (41.8–50.2)
Employed	18.8 (16.9–20.9)	15.2 (10.0–22.3)‡	24.1 (18.7–30.4)	20.9 (17.5–24.7)	11.1 (9.6–12.8)	---	11.2 (8.5–14.7)	34.6 (31.4–38.0)	33.4 (28.5–38.7)	35.4 (30.4–40.7)	38.9 (32.6–45.6)
Education level											
Not educated	8.5 (7.0–10.2)	---	---	7.4 (5.2–10.5)‡	10.5 (8.9–12.4)	---	10.3 (8.0–13.3)	40.6 (34.0–47.6)	---	44.3 (33.6–55.5)	---
Primary	16.1 (14.1 -18.3)	---	19.1 (13.3–26.5)	12.6 (10.1–15.7)	11.5 (9.9–13.3)	---	10.6 (8.1–13.7)	46.6 (42.7–50.5)	43.1 (36.4–50.1)	47.2 (41.8–52.6)	52.2 (45.3–59.0)
Secondary	16.8 (14.8–19.1)	---	16.6 (12.0 -22.6)‡	17.6 (14.9–20.7)	13.9 (11.8–16.3)	---	17.3 (14.1–21.1)	39.3 (36.4–42.2)	34.1 (29.3–39.3)	35.9 (31.9–40.1)	43.3 (39.3–47.4)
Higher	19.6 (17.3–22)	---	---	24.0 (20.1–28.5)	10.7 (7.4–15.2)	____	---	26.5 (21.7–32.0)	---	26.2 (16.8–8.5)‡	34.0 (22.0–48.5)‡
Wealth quintiles											
Q 1: Poorest	8.1 (6.4–10.2)	---	---	8.0 (5.9–10.7)	9.1 (6.5–12.5)	---	9.7 (6.0- 15.5)	56.2 (51.6–60.7)	49.2 (40.9–57.6)	52.6 (47.4–57.8)	60.0 (53.2–66.4)
Q 2	18.8 (15.5–22.5)	---	24.2 (17.4 -32.6)‡	16.4 (12.9–20.5)	11.6 (9.5–14.1)	---	14.9 (11.2–19.5)	51.7 (46.6–56.7)	45.5 (37.2–54.2)	53.8 (46.5–60.9)	56.0 (49.4–62.4)
Q 3	17.1 (14.0–20.6)	---	16.7 (11.1 -24.3)‡	20.0 (16.0–24.7)	14.9 (12.4–17.8)	---	20.5 (15.7–26.4)	41.6 (36.9–46.4)	41.4 (32.8–50.5)	42.5 (35.7–49.5)	45.7 (39.1–52.5)
Q 4	19.7 (17.1–22.8)	---	---	22.6 (18.5–27.3)	13.6 (11.1–16.6)	---	9.7 (7.3–12.8)	28.1 (23.9–32.7)	25.9 (18.5 -35.0)‡	23.0 (17.3–29.9)	29.5 (23.7–36.2)
Q5 Wealthiest	17.9 (14.9–21.3)	---	---	20.0 (15.5–25.5)	9.9 (7.2–13.3)	---	9.5 (5.8–15.3)	19.2 (14.2–25.4)	18.6 (11.7 -28.3)‡	17.2 (13.4 -21.8)‡	20.3 (13.3–29.8)
** **	**Ethiopia§**			**Kenya**				**Lesotho**			
** **	**Gen pop**	**WLHIV**	**AGYW**	**Gen pop**	**MLHIV**	**WLHIV**	**AGYW**	**Gen pop**	**MLHIV**	**WLHIV**	**AGYW**
**Coverage**	5.2 (4.5–6.0)	11.4 (8.3–15.3)	4.4 (3.6–5.3)	14.1 (13.1–15.1)	14.8 (10.4–20.7)	17.4 (14.5–20.7)	11.7 (10.1–13.6)	24.0 (22.3–25.9)	22.7 (19.8–25.9)	23.6 (21.3–26.0)	26.7 (24.2–29.3)
**Residence** Rural	5.6 (4.5–6.9)	____	5.0 (3.9–6.5)	16.2 (15.0–17.6)	13.7 (9.8–18.9)‡	19.2 (15.5–23.5)	14.6 (12.3–17.3)	32.6 (30.3–35.0)	30.7 (26.8–34.8)	33.7 (30.5–37.1)	34.7 (31.3–38.2)
Urban	4.8 (3.9–5.8)	11.4 (8.3–15.3)	3.7 (2.8–5.0)	10.8 (9.3–12.5)	---	14.4 (10.1–19.9)	7.9 (6.0–10.3)	11.9 (9.4–14.9)	11.3 (7.8–16.0)‡	11.0 (8.3–14.6)	15.6 (12.3–19.4)
Employment status											
Not employed	5.2 (4.3–6.1)	11.6 (7.8–17.0)‡	4.6 (3.7–5.7)	13.8 (12.6–15.0)	---	16.5 (12.8–20.9)	11.8 (9.9–13.8)	28.0 (26.1–30.1)	30.3 (26.3–34.6)	27.8 (24.7–31.1)	28.4 (25.6–31.3)
Employed	5.2 (4.4–6.1)	11.1 (6.9–17.2)	3.8 (2.8–5.2)‡	14.3 (13.1–15.6)	15.0 (10.4–21.3)‡	18.3 (14.2–23.3)	11.7 (9.1–14.9)	17.8 (15.9–19.9)	15.7 (12.3–19.9)	17.4 (14.7–20.5)	17.7 (13.6–22.8)
Education level											
Not educated	8.1 (6.5–10.1)	---	---	14.0 (11.7–16.7)	---	---	9.0 (5.1–15.5)‡	28.5 (24.1–33.4)	20.8 (15.2 -27.7)‡	---	---
Primary	6.3 (5.3–7.5)	14.1 (9.3–20.7)‡	4.0 (2.9–5.4)	15.3 (14.1–16.5)	17.3 (10.9–26.2)‡	15.4 (12.4–18.9)	13.0 (10.4–16.0)	29.0 (26.8–31.4)	25.5 (21.4–30.1)	28.9 (25.7–32.3)	36.2 (31.3–41.4)
Secondary	4.3 (3.5–5.2)	---	4.3 (3.3–5.5)	13.3 (12.1–14.7)	---	20.3 (14.5 -27.6)‡	11.3 (9.0–14.1)	21.1 (19.1–23.3)	20.1 (15.3–26.0)	18.3 (15.3–21.6)	23.6 (21.1–26.2)
Higher	3.2 (2.6–4.0)	---	4.8 (3.3–7.1)‡	11.4 (9.2–13.9)	---	---	10.6 (6.7–16.3)‡	15.7 (11.7–20.6)	---	---	26.4 (18.5–36.1)‡
Wealth quintiles											
Q 1: Poorest	8.2 (6.7–10.1)	---	8.1 (5.8–11.2)	17.0 (15.2–19.1)	---	17.9 (12.9 -24.2)‡	14.6 (11.2–18.7)	40.0 (36.1–44.0)	38.7 (32.2–45.7)	44.5 (38.6–50.5)	44.4 (38.7–50.2)
Q 2	4.2 (3.2–5.5)	---	---	17.3 (15.3–19.5)	---	19.6 (14.5 -26.0)‡	15.2 (11.5–19.9)	37.8 (34.2–41.4)	31.5 (25.7–38.0)	34.5 (29.8–39.6)	37.4 (32.3–42.9)
Q 3	5.7 (4-5–7.0)	---	5.1 (3.6–7.0)	17.4 (15.6–19.4)	---	18.7 (13.1 -26.0)‡	20.1 (15.2–26.1)	22.6 (19.5–26.0)	18.4 (13.6 -24.4)‡	19.6 (15.5–24.5)	26.0 (21.9–30.6)
Q 4	5.0 (3.6–6.8)	---	---	11.9 (10.1–13.8)	---	16.7 (10.8 -25.1)‡	6.9 (4.4–10.5)‡	13.5 (11.1–16.3)	---	12.9 (9.6–17.3)	13.6 (10.3–17.7)
Q5 Wealthiest	3.6 (2.6–5.0)	---	3.4 (2.3–5.2)‡	6.2 (4.6–8.3)	---	---	---	11.5 (8.4–15.6)	---	11.6 (7.9–16.7)‡	16.4 (11.5–22.9)

Gen pop is General population: MLHIV is Men living with HIV, WLHIV is women living with HIV; AGYW is adolescent girls and young women --- Results had fewer than 25 observations and are not shown. § results for MLHIV were less than 25 observations and are not shown.___ data set had no variable. ‡ Estimate based on 25–49 observations and should be interpreted with caution. Ethiopia data set had data from urban settings.

**Table 4:** Survey weighted household social protection coverage of general population, people living with HIV (male and female), adolescent girls and young women by country (PHIA 2015-2019) (percent, 95% confidence interval).

	Malawi				Namibia				Rwanda§		
	Gen pop	MLHIV	WLHIV	AGYW	Gen pop	MLHIV	WLHIV	AGYW	Gen pop	WLHIV	AGYW
General coverage	14.8 (13.6–16.2)	14.7 (11.8–18.3)	14.5 (12.4–16.9)	15.1 (13.3–17.1)	36.1 (34.3–38.0)	38.4 (33.2–43.8)	45.0 (41.2–49.0)	40.5 (37.6–43.5)	10.4 (9.3–11.5)	11.6 (8.6–15.5)	11.0 (9.7–12.5)
Residence: Rural	16.6 (15.1–18.2)	17.7 (14.0–22.1)	17.0 (14.3–20.1)	16.8 (14.7–19.1)	51.6 (48.7–54.4)	52.5 (46.3–58.7)	57.3 (53.2–61.3)	55.8 (51.8 -59.7)	11.7 (10.5–13.1)	14.4 (10.5–19.3)	12.3 (10.8–14.1)
Urban	7.8 (6.0–10.0)	---	7.9 (5.8–10.6)‡	8.5 (5.3–13.3)	25.1 (22.7–27.6)	25.2 (18.7–33.0)	32.5 (26.7–38.9)	28.4 (24.4–32.8)	5.1 (3.8–6.8)	---	6.0 (4.2–8.4)
Employment status											
Not employed	14.9 (13.5–16.4)	17.9 (13.4–23.5)	14.8 (12.2–17.8)	15.1 (13.1–17.2)	45.0 (42.8–47.2)	50.0 (43.1–56.9)	50.7 (46.5–54.8)	43.4 (40.1–46.9)	10.6 (9.4–11.8)	10.0 (6.9–14.3)‡	10.9 (9.5–12.4)
Employed	14.7 (13.1–16.4)	11.5 (8.2–16.0)‡	13.8 (9.9–18.9)‡	15.2 (11.7–19.3)	25.7 (23.8–27.6)	27.3 (21.3–34.2)	33.9 (28.1–40.3)	28.3 (23.5–33.6)	10.1 (8.9–11.4)	14.2 (9.6–20.6)‡	11.2 (9.2–13.6)
Education level											
Not educated	15.7 (13.2–18.7)	---	19.4 (13.9–6.4)‡	---	36.5 (32.7–40.4)	36.2 (24.4–50.0)‡	44.5 (33.9–55.7)	41.4 (30.7–52.9)	13.9 (12.2–5.8)	---	---
Primary	15.3 (13.9–16.9)	15.2 (11.5–19.9)	14.9 (12.2–18.1)	15.4 (13.2–17.8)	45.1 (42.2–48.0)	40.2 (33.0–47.8)	47.9 (42.0–53.8)	50.3 (45.0–55.6)	11.1 (9.9–12.4)	12.1 (8.6–16.8)‡	12.9 (11.1–14.8)
Secondary	14.1 (12.5–15.9)	---	9.9 (6.4–14.9)‡	15.6 (13.0–18.7)	35.8 (33.8–37.8)	36.0 (29.4–43.1)	44.0 (39.7–48.5)	40.2 (37.4–43.1)	8.5 (7.3–9.8)	---	9.2 (7.7–10.9)
Higher	7.8 (5.6–10.7)	---	---	---	20.1 (16.4–24.3)	---	---	23.0 (13.5 -36.2)‡	3.5 (2.2–5.4)	---	---
Wealth quintiles											
Q 1: Poorest	14.8 (12.5–17.4)	---	14.5 (9.2–22.1)‡	12.8 (9.3–17.5)	51.1 (47.6–54.7)	48.7 (40.5–56.9)	55.8 (50.1–61.4)	54.1 (49.4–58.6)	17.1 (14.9–19.5)	---	18.7 (15.1–22.8)
Q 2	16.5 (14.0–19.3)	---	19.9 (14.1–27.3)‡	16.4 (13.0–20.5)	48.7 (45.1–52.4)	51.3 (42.3–60.1)	57.5 (50.7–64.1)	56.3 (50.7–61.7)	14.2 (12.2–16.6)	---	14.0 (11.1–17.5)
Q 3	17.0 (14.5–19.8)	---	16.8 (11.6–23.6)‡	16.4 (13.2–20.3)	37.2 (33.5–41.1)	37.5 (28.7–47.1)	41.9 (35.0–49.1)	41.1 (34.9–47.6)	10.6 (8.7–12.9)	---	13.5 (10.6–17.2)
Q 4	17.2 (14.6–20.1)	---	15.8 (11.2–21.9)‡	17.3 (13.9–21.4)	24.1 (20.7–27.8)	---	23.8 (15.9–34.1)	25.6 (20.7–31.1)	8.7 (7.2–10.6)	---	8.2 (6.1–10.9)
Q5 Wealthiest	9.7 (8.0–11.7)	---	9.1 (6.2–13.0)‡	12.4 (8.9–17)	19.7 (16.2 -23.8)	---	---	22.5 (17.2–28.8)	2.6 (1.9–3.6)	---	2.5 (1.5–4.0)‡
	**Tanzania**				**Uganda§**				**Zambia**		
	**Gen pop**	**MLHIV**	**WLHIV**	**AGYW**	**Gen pop**	**WLHIV**	**AGYW**	**Gen pop**	**MLHIV**	**WLHIV**	**AGYW**
General coverage	8.8 (8.0–9.7)	10.7 (7.6–15.0)	13.8 (11.2–17.0)	8.8 (7.6–10.1)	10.4 (9.5–11.3)	10.5 (8.5–12.8)	10.9 (9.7–12.2)	7.8 (6.8–8.9)	6.3 (4-7–8.6)	7.3 (5.9–8.9)	8.1 (6.8–9.7)
Residence: Rural	9.7 (8.6–10.9)	11.6 (7.7–17.3)‡	13.6 (10.0–18.3)	10.0 (8.5–11.8)	11.0 (10.0–12.2)	11.0 (8.6–13.9)	11.9 (10.4–13.5)	9.2 (7.7–11.0)	8.5 (5.9–12.0)‡	8.9 (6.6–11.7)	9.3 (7.3–11.7)
Urban	7.4 (6.2–8.9)	---	14.1 (10.5–18.5)	7.0 (5.4–9.1)	8.8 (7.1–10.7)	9.7 (6.6–14.0)‡	8.7 (6.8–11.2)	6.1 (5.1–7.4)	---	6.2 (4.5–8.6)	6.9 (5.4–8.8)
Employment status											
Not employed	8.9 (8.0–9.9)	---	15.4 (11.9–19.8)	8.5 (7.4–9.9)	10.7 (9.7–11.9)	11.5 (8.8–14.9)	12.0 (10.5–13.6)	8.0 (6.8–9.3)	---	7.0 (5.5–8.7)	8.1 (6.7–9.7)
Employed	8.8 (7.8–9.9)	11.8 (7.8–17.6)‡	11.6 (8.3–15.9)	9.5 (7.4–12.2)	10.1 (9.2–11.1)	9.6 (7.1–12.9)	8.7 (7.2–10.6)	7.5 (6.5–8.6)	6.4 (4.3–9.3)‡	8.1 (5.6–11.5)‡	8.4 (6.2–11.3)
Education level											
Not educated	11.0 (9.1–13.1)	---	12.3 (7.2–20.3)‡	10.5 (7.4–14.7)‡	11.1 (9.0–13.5)	---	12.3 (7.7–19.2)‡	10.1 (6.8–14.6)	---	---	---
Primary	9.1 (8.1–10.1)	10.7 (7.3–15.5)‡	15.3 (12.1–19.0)	9.3 (7.7–11.1)	10.2 (9.3–11.1)	9.7 (7.4–12.6)	10.9 (9.5–12.4)	8.7 (7.3–10.2)	---	6.7 (5.1–8.9)	7.4 (5.6–9.7)
Secondary	7.8 (6.7–9.0)	---	---	8.2 (6.7–10.0)	10.9 (9.6–12.4)	---	11.1 (9.2–13.4)	7.0 (6.1–8.1)	6.0 (3.8–9.4)‡	7.1 (5.3–9.4)‡	8.2 (6.8–9.8)
Higher	4.7 (3.2–6.8)	---	_____	---	9.8 (8.4–11.5)	---	9.7 (7.1–13.1)	6.6 (4.7–9.0)	---	---	---
Wealth quintiles											
Q 1: Poorest	14.0 (11.9–16.4)	---	19.6 (13.5–27.6)‡	13.8 (10.8–17.5)	13.4 (11.9–15.0)	13.6 (9.0–19.9)‡	13.9 (12.0–16.0)	9.6 (7.3–12.7)	---	---	8.8 (5.8–13.2)
Q 2	11.0 (9.2–13.0)	---	16.5 (10.9–24.1)‡	10.9 (8.1–14.4)	11.3 (9.4–13.5)	---	12.3 (9.9–15.2)	7.5 (6.1–9.3)	---	---	8.3 (6.0–11.5)
Q 3	8.3 (7.0–9.9)	---	14.7 (10.5–20.3)‡	10.5 (7.8–14.0)	9.5(7.6–11.7)	---	10.2 (7.5–13.8)	10.6 (8.3–13.4)	---	7.5 (4.8–11.8)‡	9.0 (6.4–12.5)
Q 4	6.9 (5.3–8.9)	---	---	7.0 (5.0–9.8)	10.7 (8.7–13.1)	11.8 (8.3–16.6)‡	10.8 (8.3 -14.0)	6.1 (4.9–7.5)	---	6.0 (4.1–8.7)‡	7.8 (5.6–10.9)
Q5 Wealthiest	4.4 (3.3–5.9)	---	---	3.5 (2.1–5.8)‡	7.1 (5.6–8.9)	---	7.5 (5.5–10.3)	6.3 (4.8–8.1)	---	6.6 (4.4–9.9)‡	7.3 (5.3–9.9)

Gen pop General population: MLHIV Men living with HIV, WLHIV women living with HIV; AGYW adolescent girls and young women --- Results had fewer than 25 observations and are not shown. § Results had fewer than 25 observations and are not shown for MLHIV including in Uganda where general coverage was 11.5 (8.8–14.8), for rural 11.5 (8.4–15.6) employed 12.2 (8.9–16.4) and primary education). ___ data set had no variable. ‡ Estimate based on 25–49 observations and should be interpreted with caution.

**Table 5:** Survey weighted household social protection coverage of the general population, people living with HIV (male and female), and adolescent girls and young women by country (PHIA 2015-2019) (percent, 95% confidence interval).

	Zimbabwe			
	Gen pop	MLHIV	WLHIV	AGYW
General coverage	19.9 (18.6–21.2)	18.6 (16.1–21.3)	18.4 (16.4–20.5)	20.0 (18.3–21.8)
Residence: Rural	26.8 (24.9–28.7)	25.2 (21.8–28.9)	25.4 (22.6–28.4)	27.1 (24.6–29.8)
Urban	7.6 (6.2–9.2)	---	7.1 (5.0–10.0)	8.9 (7.1–11.1)
Employment status				
Not employed	21.7 (20.3–23.1)	20.8 (17.1–25.1)	18.5 (16.3–21.0)	20.7 (18.8–22.7)
Employed	17.2 (15.7–18.7)	16.4 (13.5–19.8)	18.1 (14.8–21.9)	17.6 (14.7–20.8)
Education level				
Not educated	23.0 (19.1–27.4)	---	---	---
Primary	22.3 (21.1–24.8)	16.8 (12.9–21.6)	20.4 (17.6–23.5)	20.9 (17.5–24.8)
Secondary	19.7 (18.3–21.2)	19.6 (16.5–23.1)	17.6 (15.0–20.6)	20.2 (18.3–22.3)
Higher	10.9 (8.7–13.4)	---	---	12.9 (7.8–20.6)‡
**Wealth quintiles**				
Q 1: Poorest	30.7 (27.9–33.7)	27.8 (22.0–34.5)	30.9 (26.2–36.0)	30.7 (26.4–35.3)
Q 2	32.1 (29.4–34.9)	32.5 (26.2–39.5)	28.4 (23.3–34.2)	32.5 (28.4–36.8)
Q 3	22.8 (20.0–25.9)	18.8 (13.0–26.4)‡	22.6 (17.2–29.0)	23.4 (19.5–27.8)
Q 4	7.5 (5.8–9.5)	---	5.6 (4.0–8.8)‡	8.3 (5.9–11.5)
Q5 Wealthiest	8.1 (6.4–10.2)	---	7.8 (5.2–11.3)‡	9.9 (7.6–12.7)

Gen pop General population: MLHIV Men living with HIV, WLHIV women living with HIV; AGYW adolescent girls and young women --- Results had fewer than 25 observations and were suppressed. ___ data set had no variable. ‡ Estimate based on 25–49 observations and should be interpreted with caution.

**Fig 1:**
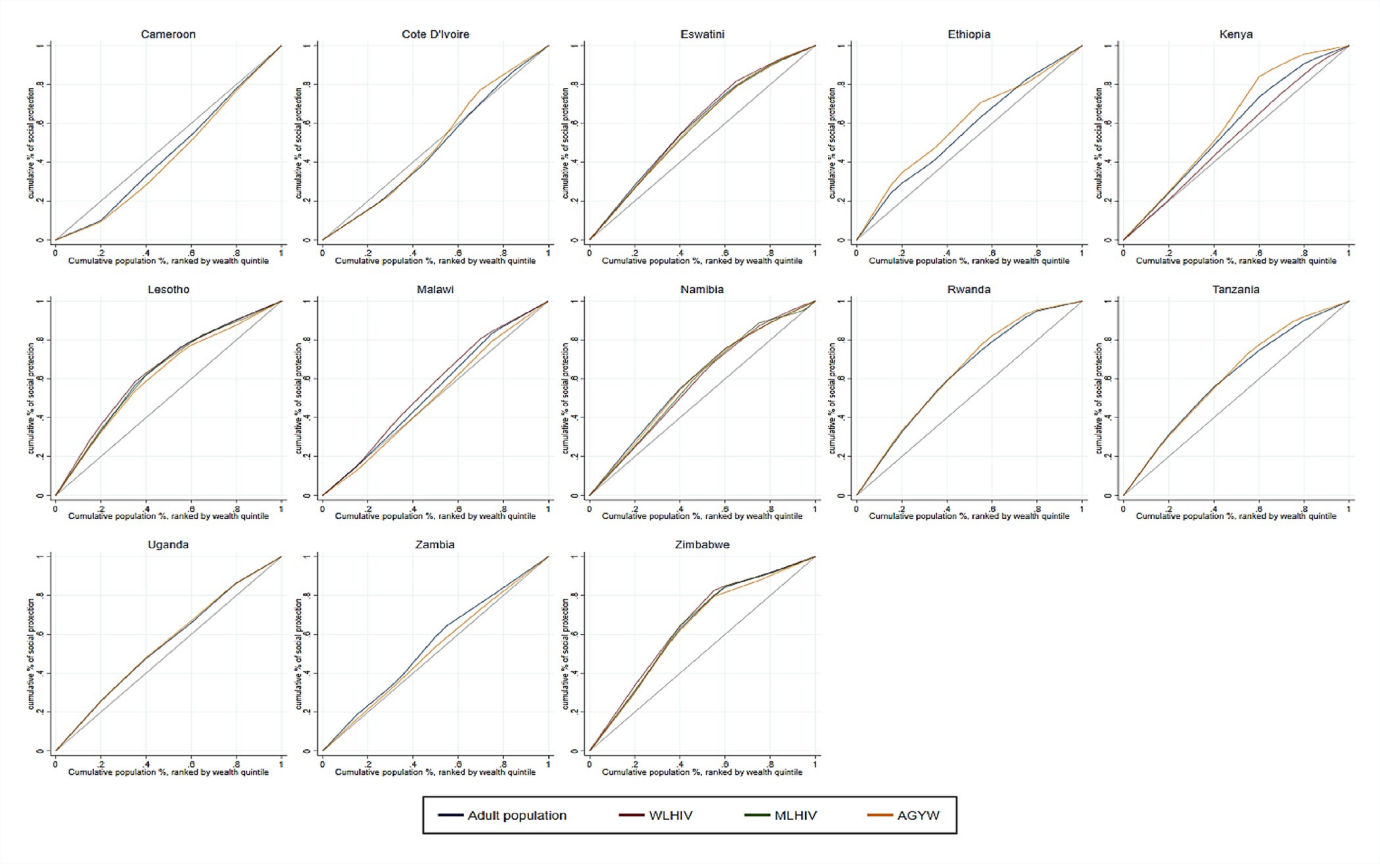
Concentration curves of receiving social protection among the general population, people living with HIV (women and men), and adolescent girls and young women in sub-Saharan African Countries (PHIA 2015-2019).

Fig 1 presents the concentration curves of access to social protection by country for the general population, MLHIV and WLHIV, and AGYW. Table 6 reports the associated concentration indices. The results show that socioeconomic inequalities in access to social protection were pro-rich only in Cameroon, among the general population, and AGYW–evident as the concentration curves lie below the line of equality—with a CIX value of 0.122 (p < 0.001) among the general population and a CIX value of 0.169 (<0.001) among AGYW. This result shows that more people from wealthier than poor households reported receiving social protection. In Côte d’Ivoire, socioeconomic inequalities in receiving social protection were pro-rich, but the associated CIX estimates were not significantly different from zero. In the remaining 11 countries, social protection was pro-poor, in line with our initial hypothesis. The CIX values for socioeconomic inequalities in receiving social protection in these countries ranged from −0.080 (p = 0.002) among the general population in Malawi to −0.372 (p < 0.001) among WLHIV in Zimbabwe (Table 6).

**Table 6:** Survey weighted concentration index of socioeconomic inequalities in receiving social protection for the general population, people living with HIV (male and female) and adolescent girls and young women (CIX, p-value, sample size), PHIA 2015-2019.

** **	**Cameroun**			**Cote D’Ivoire**			**Eswatini**			**Ethiopia**			**Kenya**			**Lesotho**			**Malawi**		
** **	**CIX**	**p-value**	**sample size**	**CIX**	**p-value**	**sample size**	**CIX**	**p-value**	**sample size**	**CIX**	**p-value**	**sample size**	**CIX**	**p-value**	**sample size**	**CIX**	**p-value**	**sample size**	**CIX**	**p-value**	**sample size**
Gen population	0.122	<0.001	24850	0.046	0.234	17268	-0.320	<0.001	9523	-0.133	0.001	18449	-0.175	<0.001	21409	-0.351	<0.001	11640	-0.080	0.002	16685
WLHIV	---	---	---	---	---	---	-0.326	<0.001	1909	---	---	---	-0.072	0.173	1016	-0.369	<0.001	2176	-0.128	0.012	1475
MLHIV	---	---	---	---	---	---	-0.271	<0.001	867	---	---	---	---	---	---	-0.335	<0.001	1016	---	---	---
AGYW	0.169	<0.001	4936	0.010	0.848	3163	-0.324	<0.001	1922	-0.196	0.001	4773	-0.249	<0.001	2587	-0.315	<0.001	2350	-0.017	0.658	3578
	**Namibia**			**Rwanda**			**Tanzania**			**Uganda**			**Zambia**			**Zimbabwe**					
	**CIX**	**p-value**	**sample size**	**CIX**	**p-value**	**sample size**	**CIX**	**p-value**	**sample size**	**CIX**	**p-value**	**sample size**	**CIX**	**p-value**	**sample size**	**CIX**	**p-value**	**sample size**			
Gen population	-0.301	<0.001	16220	-0.299	<0.001	29435	-0.229	<0.001	28289	-0.115	<0.001	27868	-0.095	0.008	18991	-0.353	<0.001	19612			
WLHIV	-0.291	<0.001	1622	---	---	---	---	---	---	---	---	---	---	---	---	-0.372	<0.001	2151			
MLHIV	-0.275	<0.001	713	---	---	---	---	---	---	---	---	---	---	---	---	-0.347	<0.001	1084			
AGYW	-0.306	< 0.001	3007	-0.320	<0.001	6063	-0.249	<0.001	5824	-0.121	0.001	6315	-0.043	0.340	4144	-0.331	<0.001	3927			

---The results for the following countries and subpopulations were not shown because their observations for the wealth variables were fewer than 25: Cameroon, Côte d’Ivoire, Ethiopia, Rwanda, Tanzania, Uganda, and Zambia for MLHIV and WLHIV; Kenya and Malawi for MLHIV.

In Eswatini, Lesotho, Rwanda, Namibia, and Zimbabwe, socioeconomic inequalities in receiving social protection were pro-poor, below −0.300, among all population groups. Socioeconomic inequalities in receiving social protection access were pro-poor and moderate, with CIX values ranging from −0.100 to −0.300 among the general population and AGYW in Ethiopia, Kenya, and Uganda and among the general population, WLHIV, and AGYW in Tanzania. Social protection was pro-poor and of low inequality, that is, between CIX −0.010 and CIX = −0.100 among the general population and MLHIV and WLHIV in Malawi, and the general population and MLHIV in Zambia (Fig 1 and Table 6).

## 4 Discussion

This study examined economic-related inequality in receiving social protection among the general population, MLHIV and WLHIV, and AGYW in 13 sub-Saharan African countries. The study also evaluated whether people in the poorest households received social protection. Our findings showed that the proportion of the general population receiving social protection varied from 5.2% (95% CI 4.5%–6.0%) in Ethiopia to 39.9% (37.0%–42.8%) in Eswatini. Social protection was pro-poor in 11 out of the 13 countries studied, implying that more people from poor households received social protection than those from wealthier households in these 11 countries. However, in eight of these 11 countries, less than 15% of people from the poorest quintile households reported receiving social protection. Cameroon was the only country where social protection was pro-rich. These results bear considerable policy implications for the targeting, scale up, and equalization of access to social protection among the general population, MLHIV and WLHIV, and AGYW.

The results of our study confirm that the proportion of respondents receiving social protection varied across groups within countries and between countries. It ranged from 4.4% among AGYW in Ethiopia to 44.6% among WLHIV in Namibia. Eswatini, Namibia and Lesotho have the highest social protection coverage, in contrast Ethiopia, Tanzania and Zambia which have the lowest. Lesotho and Namibia finance social protection from domestic tax revenues [22] than the other countries surveyed. Countries in stable financial situation can provide better and more stable social protection. These results align with previous research that has documented a broad variation in social protection coverage throughout sub-Saharan Africa. The International Labor Organization (ILO) report, 2020–2022 indicated that only 46.9% of the global population were covered by at least one social protection benefit in 2020 [22]. The ILO report further highlighted considerable regional disparities in access to social protection, with the lowest coverage in Africa at 17.4% and the highest in Europe and Central Asia at 83.9% [22]. A study covering Eswatini, Malawi, Tanzania, and Zambia found that the proportion receiving social protection varied from 7.7% in Zambia to 39.6% in Eswatini [23]. The paper found comparable social protection coverage among the AGYW and PLHIV to the general population in Malawi and Zambia [23]. This result suggests countries that finance social protection from domestic revenues can provide higher coverage of social protection, reflecting political commitment. International aid may help expand coverage to population groups excluded from social protection. Enhanced collaboration between governments, international agencies, and non-governmental and civil society organizations could improve the design and implementation of inclusive and adequate social protection systems.

Our second finding revealed that social protection was pro-poor in 11 of the 13 countries surveyed. The pro-poor social protection found in our study was expected and aligns with our hypothesis. Social protection programs are generally focused on the most impoverished households [11,16–18]. In this regard, our findings are consistent with the core objectives of social protection, which prioritize the poorest households. However, in eight of these 11 countries, fewer than 15% of people from households in the bottom wealth quintile reported receiving social protection. A review of The Atlas of Social Protection Indicators of Resilience and Equity data of the World Bank covering 123 countries conducted in 2020 found that only 22% of the poorest 20% received social assistance, which aligns with our finding [24]. Cameroon stood out as an outlier, exhibiting pro-rich social protection, underscoring a significant shortfall in reaching people from the poorest households. A contributing factor to this disparity in Cameroon was the high access to social protection among employed individuals, indicating that the benefits were linked to their employment. For example, civil service pensions, which benefited only 141,000 pensioners in 2016 in Cameroon, were allocated over 10 times more funding by the government of Cameroon than all social assistance schemes combined [25]. Another potential reason was the relatively nascent state of social protection in Cameroon (Levine, Socpa, Both, Salomon, & Fomekong, 2022). Despite the development of a comprehensive social protection policy in 2017, the program was not approved. Social protection programs remained small-scale and uncoordinated [25]. This result shows a potential gap in reaching people from the poorest households.

The limited coverage of individuals from the poorest quintile households identified in our study may be due to difficulties faced by low-income countries in identifying the poorest population groups to target their social protection services [24]. Another reason may be the dynamic mobility of people across economic groups. For example, in the Occupied Palestine State, where 40% of individuals receiving social protection were categorized as poor, they moved up and down income groups over time [26]. These findings underscore that pro-poor social protection alone is insufficient to reach people from the poorest households. Although policymakers may contemplate redistributing social protection benefits from wealthier to poor households, this approach may not be feasible or desirable. Wealthier households can descend into poverty, requiring social protection [26]. Another potential explanation could be the non-take-up of social protection benefits, a common phenomenon among marginalized populations who need social protection the most [27]. Non-take-up pertains to eligible individuals not accessing available benefits for a range of reasons, including lack of information, complex or costly procedures, limited access to digital technology and know-how, stigma, discrimination, shame, and fear of interacting with social services [27]. Moreover, people from the poorest households, eligible to access social protection, may not take up available social protection benefits owing to inadequate coverage and the narrow scope of programs [28]. According to our study, only in countries with an overall higher social protection coverage, such as Eswatini, Lesotho, Namibia, and Zimbabwe, the proportion of the poorest wealth quintile households reached were also high. A study examining global inequalities in accessing reproductive, maternal, newborn, and child health services showed that countries, with low inequality and high coverage in these services, effectively reached the poorest women and children [29]. The coverage of social protection needs to be broadened and deepened to reach the poorest households. Additionally, strategies to identify households that are thrust into poverty owing to emerging risks, such as financial crises, conflicts, droughts, disasters, and pandemics like COVID-19, and link them to social protection, should be developed.

In our study, the concentration curves and CIX indicate inequalities in receiving social protection among the public, PLHIV, and AGYW. The direction and magnitude of inequalities were similar between population groups. In Eswatini, Lesotho, Namibia, Rwanda, and Zimbabwe, major socioeconomic inequalities favoring the poor were observed in all the population groups. In other countries the inequalities were low among all the population groups also favoring the poor except in Cameroon. The high pro-poor inequality in receiving social protection among all the population groups in Zimbabwe shows that policymakers can also work with moderate social protection coverage to reach poor households. These results suggest that inequalities in receiving social protection discriminated in favor of all population groups regardless of the direction and magnitude of the inequalities. The public, PLHIV and AGYW, among the poor or the wealthier, in general had similar coverage of social protection. This crucial finding underscores the need for pro-poor and inclusive social protection policies.

This study has several limitations and strengths. Contrary to the ILO’s strategy of presenting summarized national responses to government-provided social protection [22], our study compiles individual responses from various countries through household surveys. Notwithstanding, our estimates correspond to the data from the ILO 2020–2022 report, indicating that our measurement reflects the same information that governments use in their reporting. Another limitation of our study is the absence of identification for marginalized people, such as gay men and other men who have sex with men, sex workers and migrants. Marginalized population groups suffer a bulk of hardships owing to inequalities [1,2]. These population groups may be excluded from accessing social protection benefits often because of stigma, discrimination, and punitive laws [30]. The barriers to accessing social protection may be more pronounced among adolescent and young key populations. Concrete suggestions on how to reach young key populations with social protection are difficult to formulate without understanding the key barriers they face in accessing social protection. The barriers to accessing social protection benefits of these subgroups were not addressed in this study. This gap stems from either a lack of available information or an insufficient sample size to conduct meaningful analysis. Furthermore, our analysis did not differentiate among the specific types of social protection benefits received or their monetary value, nor did we track changes in social protection coverage over time. This was because we relied on cross-sectional survey data that only captured access to social protection in the preceding 12 months of the survey. Moreover, as the data were collected before 2020, the rapid expansion of social protection measures in response to the COVID-19 pandemic was also not captured. Future research should examine the evolution of social protection coverage over time among different population subgroups in sub-Saharan Africa. It should also investigate the state of social protection post the COVID-19 pandemic and assess the factors contributing to observed changes.

## 5. Conclusion

In the countries surveyed, access to social protection for the general population, MLHIV and WLHIV, and AGYW was low but favored people from poor households in majority of the countries studied. However, pro-poor social protection, although necessary, is not sufficient to ensure that people from the poorest households receive social protection. Further research is required to identify and reach people from the poorest households with social protection in sub-Saharan Africa.

## Supporting information

S1 Table Population HIV impact assessment country reports. (DOCX)

S2 Table Variable descriptions. (DOCX)

S3 Table Survey weighted proportions of men and women living with HIV by country (PHIA 2015-2019). (DOCX)
