# Supplementary material for: Missing the vulnerable—Inequalities in social protection in 13 sub-Saharan African countries: Analysis of population-based surveys
Source: PLOS Glob Public Health. 2024 Jul 2;4(7):e0002973. doi: 10.1371/journal.pgph.0002973 (PMC11218938; doi:10.1371/journal.pgph.0002973)
Supplement: S1 Table — (DOCX) [file pgph.0002973.s001.docx]

S1 Table: Population HIV Impact Assessment Country Reports

Cameroun:

Ministry of Health (MOH), Division of Health Operations Research (DROS). Cameroon Population-based HIV Impact Assessment (CAMPHIA) 2017-2018: Final Report. Yaounde: MOH, DROS; December 2020.

Côte d'Ivoire:

Ministère de la Santé et de l’Hygiène Publique (MSHP). Côte d’Ivoire Population-Based HIV Impact Assessment (CIPHIA) 2017-2018: Final Report. Abidjan: MSHP; March 2021.

Ethiopia:

Ethiopian Public Health Institute (EPHI). Ethiopia Population-based HIV Impact Assessment (EPHIA) 2017-2018: Final Report. Addis Ababa: EPHI; August 2020.

Eswatini:

Government of the Kingdom of Eswatini. Swaziland HIV Incidence Measurement Survey 2 (SHIMS2) 2016-2017. Final Report. Mbabane: Government of the Kingdom of Eswatini; April 2019.

Kenya:

National AIDS and STI Control Programme (NASCOP). Kenya Population-based HIV Impact

Assessment (KENPHIA) 2018: Final Report. Nairobi: NASCOP; August 2022.

Lesotho:

Ministry of Health, Lesotho, Centers for Disease Control and Prevention (CDC), and ICAP at Columbia University. Lesotho Population-based HIV Impact Assessment (LePHIA) 2016-2017: Final Report. Maseru, Lesotho, Atlanta, Georgia, and New York, New York, USA: Ministry of Health, CDC, and ICAP, September 2019.

Malawi:

Ministry of Health, Malawi. Malawi Population-Based HIV Impact Assessment (MPHIA) 2015-2016: Final Report. Lilongwe, Ministry of Health. October 2018.

Namibia:

Ministry of Health and Social Services (MoHSS), Namibia. Namibia Population-based HIV Impact Assessment (NAMPHIA) 2017: Final Report. Windhoek: MoHSS, Namibia; November, 2019.

Rwanda:

Rwanda Biomedical Center (RBC). Rwanda Population-Based HIV Impact Assessment

(RPHIA) 2018-2019: Final Report. Kigali: RBC; September 2020.

Tanzania:

Tanzania Commission for AIDS (TACAIDS), Zanzibar AIDS Commission (ZAC). Tanzania HIV Impact Survey (THIS) 2016-2017: Final Report. Dar es Salaam, Tanzania. December 2018.

Uganda:

Ministry of Health, Uganda. Uganda Population-based HIV Impact Assessment (UPHIA) 2016-2017: Final Report. Kampala: Ministry of Health; July, 2019.

Zambia

Ministry of Health, Zambia. Zambia Population-based HIV Impact Assessment (ZAMPHIA) 2016: Final Report. Lusaka, Ministry of Health. February 2019.

Zimbabwe

Ministry of Health and Child Care (MOHCC), Zimbabwe. Zimbabwe Population-based HIV Impact Assessment (ZIMPHIA) 2015-2016: Final Report. Harare: MOHCC; August 2019.
