# Supplementary material for: Missing the vulnerable—Inequalities in social protection in 13 sub-Saharan African countries: Analysis of population-based surveys
Source: PLOS Glob Public Health. 2024 Jul 2;4(7):e0002973. doi: 10.1371/journal.pgph.0002973 (PMC11218938; doi:10.1371/journal.pgph.0002973)
Supplement: S2 Table — (DOCX) [file pgph.0002973.s002.docx]

S2 Table: Variable descriptions.

| Variable name | Description | Measure |
| --- | --- | --- |
| Social protection | Acknowledging receiving any economic support, including assistance for school fees, material support for education, food assistance, support for income generation, social pensions, and cash transfers including pensions, disability and child grants was recorded as receiving social protection benefits from the household interview questionnaire. | Binary |
| HIV prevalence | The proportion of people 15 years or older reporting testing HIV positive and laboratory confirmed HIV test results from the adult biomarker questionnaire. | Binary |
| Sex | Self-reported sex (i.e., male, female), from the adult interview questionnaire. | Binary |
| Age | Self-reported linear years categorised in years (15–24, 25–34, 35–44, 45–54 and 55 and above), from the adult interview questionnaire. | Categorical |
| Residence type | Self-reported residence (i.e., rural, urban), from the household questionnaire. | Binary |
| Marital status | Self- reported marriage status, i.e., married including cohabiting from the adult interview questionnaire. | Binary |
| Household size | Number of household members, reported by the head of household from the household questionnaire categorized 1 – 3, 4-6, and > 7. | Categorical |
| Employment status | Combined reported working in the past 12 months and/or past seven days from the questions "Have you done any work in the last 12 months for which you received a salary, cash, or in kind as payment?" and "Have you done any work in the last 7 days for which you received a salary, cash or in-kind as payment?" from the adult interview questionnaire. | Binary |
| Regions | A political area, often the primary sampling unit such as a province or region in some countries. | Categorical |
| Educational status | Level of school that respondent had ever attended (i.e., not educated, primary school education, secondary, higher education), from the adult interview questionnaire. | Categorical |
| Wealth | Assessed household wealth with the wealth quintiles variable, from a wealth index. Wealth quintiles ranked households depending on their household characteristics and asset ownership from Q1 representing the poorest households; to Q5 the wealthiest. (i.e., poorest, poor, rich, richer, richest). The variable was provided the PHIA dataset. | Ordered ranking |

| Supplementary Table 3: Survey weighted proportions of men and women living with HIV by country (PHIA 2015-2019). (The results are reported as (percentages with, sample size, 95% confidence intervals and absolute numbers) | | | |
| --- | --- | --- | --- |
| Country | Men and Women | Men | Women |
| Cameroon (N=26039) | 3.6 (3.3 - 4.0) 924 | 30.7 (27.5 - 34.1) 266 | 69.3 (65.9 - 72.5) 658 |
| Côte D'Ivoire (N=18339) | 2.7 (2.4 - 3.1) 417 | 30.3 (25.4 - 35.7) 124 | 69.7 (64.3 - 74.6) 293 |
| Eswatini (N=10197) | 27.9 (26.5 - 29.3) 2776 | 33.2 (31.8 - 34.7) 867 | 66.8 (65.3 - 68.2) 1909 |
| Ethiopia (N=18466) | 3.0 (2.6 - 3.4) 588 | 31.9 (27.9 - 36.1) 144 | 68.1 (63.9 - 72.1) 444 |
| Kenya (N=23536) | 5.8 (5.4 - 6.3) 1387 | 30.8 (28.2 - 33.7) 371 | 69.2 (66.3 - 71.8) 1016 |
| Lesotho (N=12842) | 25.6 (24.7 - 26.5) 3192 | 40.7 (39.2 - 42.2) 1016 | 59.3 (57.8 - 60.8) 2176 |
| Malawi (N=19092) | 10.5 (9.9 - 11.2) 2155 | 39.0 (36.7 - 41.4) 680 | 61.0 (58.6 - 63.3) 1475 |
| Namibia (N=18009) | 12.5 (11.7 - 13.4) 2335 | 35.3 (33.6 - 37.1) 713 | 64.7 (62.9 - 66.4) 1622 |
| Rwanda (N=29510) | 3.0 (2.6 - 3.3) 886 | 35.4 (32.3 - 38.7) 284 | 64.6 (61.3 - 677) 602 |
| Tanzania (N=29577) | 5.0 (4.7 - 5.4) 1707 | 33.9 (31.1 - 36.9) 517 | 66.1 (63.1 - 68.9) 1190 |
| Uganda (N=28212) | 6.3 (5.9 - 6.7) 1700 | 35.7 (33.8 - 37.7) 542 | 64.3 (62.3 - 66.2) 1158 |
| Zambia (N=21138) | 12.0 (11.3 - 12.6) 2447 | 37.6 (35.9 - 39.4) 770 | 62.4 (60.6 - 64.1) 1677 |
| Zimbabwe (N=21424) | 14.1 (13.4 - 14.8) 3235 | 40.0 (38.4 - 41.7) 1084 | 60.0 (58.3 - 61.6) 2151 |
