# Supplementary material for: Missing the vulnerable—Inequalities in social protection in 13 sub-Saharan African countries: Analysis of population-based surveys
Source: PLOS Glob Public Health. 2024 Jul 2;4(7):e0002973. doi: 10.1371/journal.pgph.0002973 (PMC11218938; doi:10.1371/journal.pgph.0002973)
Supplement: S3 Table — (DOCX) [file pgph.0002973.s003.docx]

| S3 Table: Survey weighted proportions of men and women living with HIV by country (PHIA 2015-2019). (The results are reported as (percentages with, sample size, 95% confidence intervals and absolute numbers) | | | |
| --- | --- | --- | --- |
| Country | Men and Women | Men | Women |
| Cameroon (N=26039) | 3.6 (3.3 - 4.0) 924 | 30.7 (27.5 - 34.1) 266 | 69.3 (65.9 - 72.5) 658 |
| Côte D'Ivoire (N=18339) | 2.7 (2.4 - 3.1) 417 | 30.3 (25.4 - 35.7) 124 | 69.7 (64.3 - 74.6) 293 |
| Eswatini (N=10197) | 27.9 (26.5 - 29.3) 2776 | 33.2 (31.8 - 34.7) 867 | 66.8 (65.3 - 68.2) 1909 |
| Ethiopia (N=18466) | 3.0 (2.6 - 3.4) 588 | 31.9 (27.9 - 36.1) 144 | 68.1 (63.9 - 72.1) 444 |
| Kenya (N=23536) | 5.8 (5.4 - 6.3) 1387 | 30.8 (28.2 - 33.7) 371 | 69.2 (66.3 - 71.8) 1016 |
| Lesotho (N=12842) | 25.6 (24.7 - 26.5) 3192 | 40.7 (39.2 - 42.2) 1016 | 59.3 (57.8 - 60.8) 2176 |
| Malawi (N=19092) | 10.5 (9.9 - 11.2) 2155 | 39.0 (36.7 - 41.4) 680 | 61.0 (58.6 - 63.3) 1475 |
| Namibia (N=18009) | 12.5 (11.7 - 13.4) 2335 | 35.3 (33.6 - 37.1) 713 | 64.7 (62.9 - 66.4) 1622 |
| Rwanda (N=29510) | 3.0 (2.6 - 3.3) 886 | 35.4 (32.3 - 38.7) 284 | 64.6 (61.3 - 677) 602 |
| Tanzania (N=29577) | 5.0 (4.7 - 5.4) 1707 | 33.9 (31.1 - 36.9) 517 | 66.1 (63.1 - 68.9) 1190 |
| Uganda (N=28212) | 6.3 (5.9 - 6.7) 1700 | 35.7 (33.8 - 37.7) 542 | 64.3 (62.3 - 66.2) 1158 |
| Zambia (N=21138) | 12.0 (11.3 - 12.6) 2447 | 37.6 (35.9 - 39.4) 770 | 62.4 (60.6 - 64.1) 1677 |
| Zimbabwe (N=21424) | 14.1 (13.4 - 14.8) 3235 | 40.0 (38.4 - 41.7) 1084 | 60.0 (58.3 - 61.6) 2151 |
